# Sleeve basal segmentectomy with pulmonary artery division for lung cancer: how to do it

**DOI:** 10.1007/s00595-025-03007-z

**Published:** 2025-02-07

**Authors:** Yojiro Yutaka, Taiki Ryo, Hiroshi Date

**Affiliations:** Department of Thoracic Surgery, Kyoto University Graduate School of Medicine, Kyoto University Hospital, 54 Kawahara-Cho, Shogoin, Sakyo-Ku, Kyoto, 606-8507 Japan

**Keywords:** Sleeve basal segmentectomy, Lung cancer, Double-barrel

## Abstract

Sleeve segmentectomy was originally developed to preserve the lung volume in patients who were unable to tolerate lobectomy. We herein report an immunocompromised patient with potentially curable peripheral squamous cell carcinoma in whom sleeve basal segmentectomy was performed for a 1.8-cm tumor with endobronchial growth that occluded B7 and invaded the inlet of the middle lobar bronchus and B6. To provide sufficient surgical exposure for complex double-barrel reconstruction after segmentectomy, A6 was divided and reconstructed after the anastomosis was complete. This technique may be applicable to other peripheral bronchial reconstructions to minimize blood flow disruption to bronchial anastomosis.

**Supplementary Information:**

The online version contains supplementary material available at 10.1007/s00595-025-03007-z.

## Introduction

Sleeve segmentectomy with double-barrel reconstruction is challenging because of the proximity of the surrounding pulmonary vessels [1]. While extensive detachment of the peribronchial tissue is often required to obtain an adequate surgical view for bronchial reconstruction, this approach can compromise blood flow to the bronchial anastomosis, increasing the risk of ischemia [2].

We herein report a case of sleeve basal segmentectomy during which A6 was divided to ensure an adequate view rather than performing extensive peribronchial tissue detachment. Once double-barrel anastomosis was completed, A6 was reconstructed.

## Case presentation

### Medical history

**Fig. 1:**
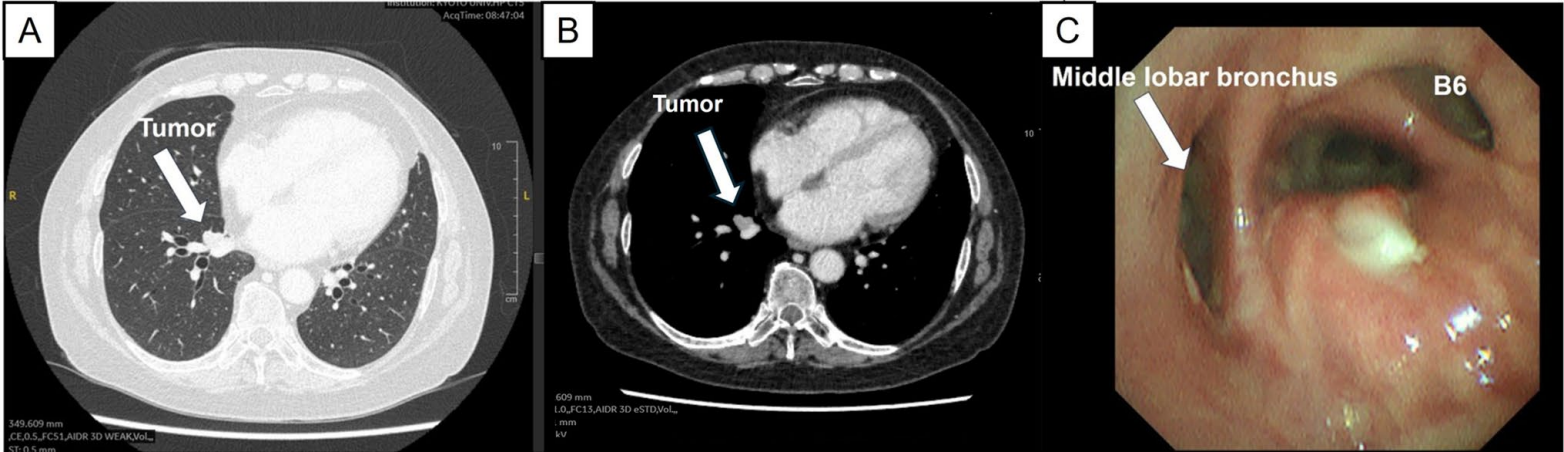
Preoperative findings. **A**, **B** Chest computed tomography showed a 1.8-cm tumor in S7. **C** Bronchoscopy revealed complete occlusion of B7 by the tumor, which had invaded the bronchus intermedius

A 64-year-old female former smoker presented with a lung mass. She had a history of rheumatoid arthritis and had been receiving long-term steroid and biological treatment. Chest computed tomography (CT) revealed a 1.8-cm tumor occluding B7 and a 1.7-cm cavity with an irregularly thickened wall in segment 2 of the right upper lobe. Staging positron emission tomography/CT showed maximum standardized uptake values of 5.6 in the S7 tumor and 4.7 in the S2 tumor without evidence of regional lymphadenopathy (Fig. 1A, B). Bronchoscopy confirmed that B7 was completely occluded by a tumor that invaded the bronchus intermedius, resulting in a diagnosis of double squamous cell carcinoma (cT1bN0M0: S2 and S7; Fig. 1C). Preoperative respiratory function tests were as follows: vital capacity, 2810 mL (83.3%); forced expiratory volume in 1 s, 1900 mL (84.4%); and percent predicted diffusing capacity of the lung for carbon monoxide, 63.5%. Because chest CT suggested slight emphysematous and fibrotic changes and the patient had rheumatoid arthritis, we were concerned about the possibility of interstitial pneumonia. Therefore, we recommend surgical resection rather than radiotherapy. Given the proximity of the tumor to the orifices of the middle lobar bronchus and B6, we planned sleeve basal segmentectomy combined with S2 wedge resection to preserve the middle lobe and S6.

### Surgical techniques and operative findings

**Fig. 2:**
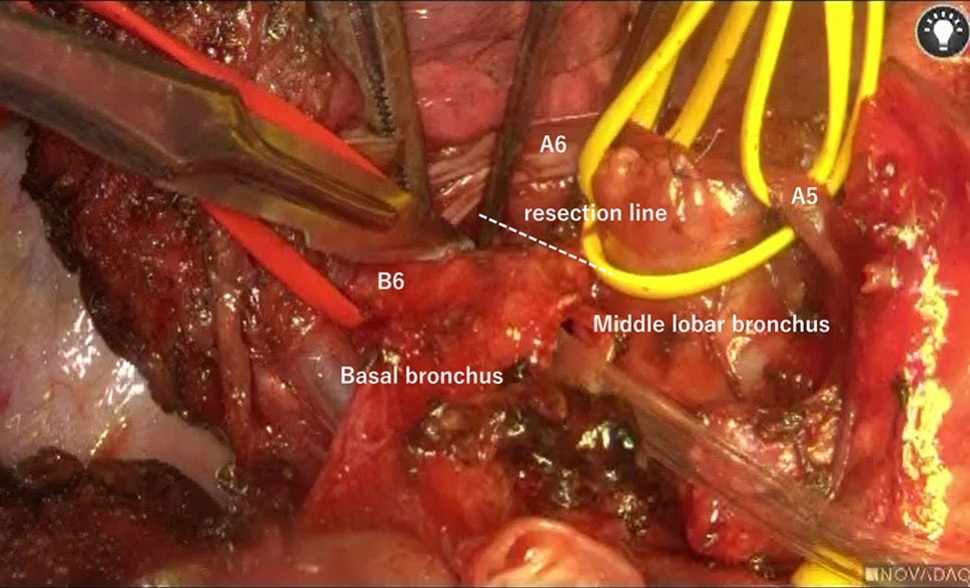
Intraoperative findings before cutting the basal bronchus. The basal bronchus was carefully transected using a sharp scalpel while maintaining a gross surgical margin of at least 1 cm

After obtaining written informed consent from the patient, the S2 cavitary lesion was first removed through posterolateral thoracotomy in the fifth intercostal space. The tumor was palpable as an elastic soft mass. To minimize pulmonary resection, electrocautery was used to outline the planned resection line on the pleural surface; the line accounted for the tumor plus a 10-mm surgical margin. The pulmonary parenchyma was gradually divided, and the deepest portion of the tumor was resected using a linear stapler. Next, we performed basal sleeve segmentectomy. After exposing the interlobar pulmonary artery, the basal artery was divided and closed with a running 6–0 polypropylene suture, while the interlobar artery was controlled proximally using a vascular clamp. The indocyanine green technique was then used to demarcate the segmental line, facilitating division of the S6 and basal segments with a stapler. The hilar lymph nodes (#7, 10, 11s, and 12l) were dissected. The proximal and distal transection lines at the bronchus were carefully determined, and the middle lobar bronchus, B6, and bronchus intermedius were transected using a sharp scalpel, maintaining at least a 1-cm gross surgical margin (Fig. 2).

**Fig. 3:**
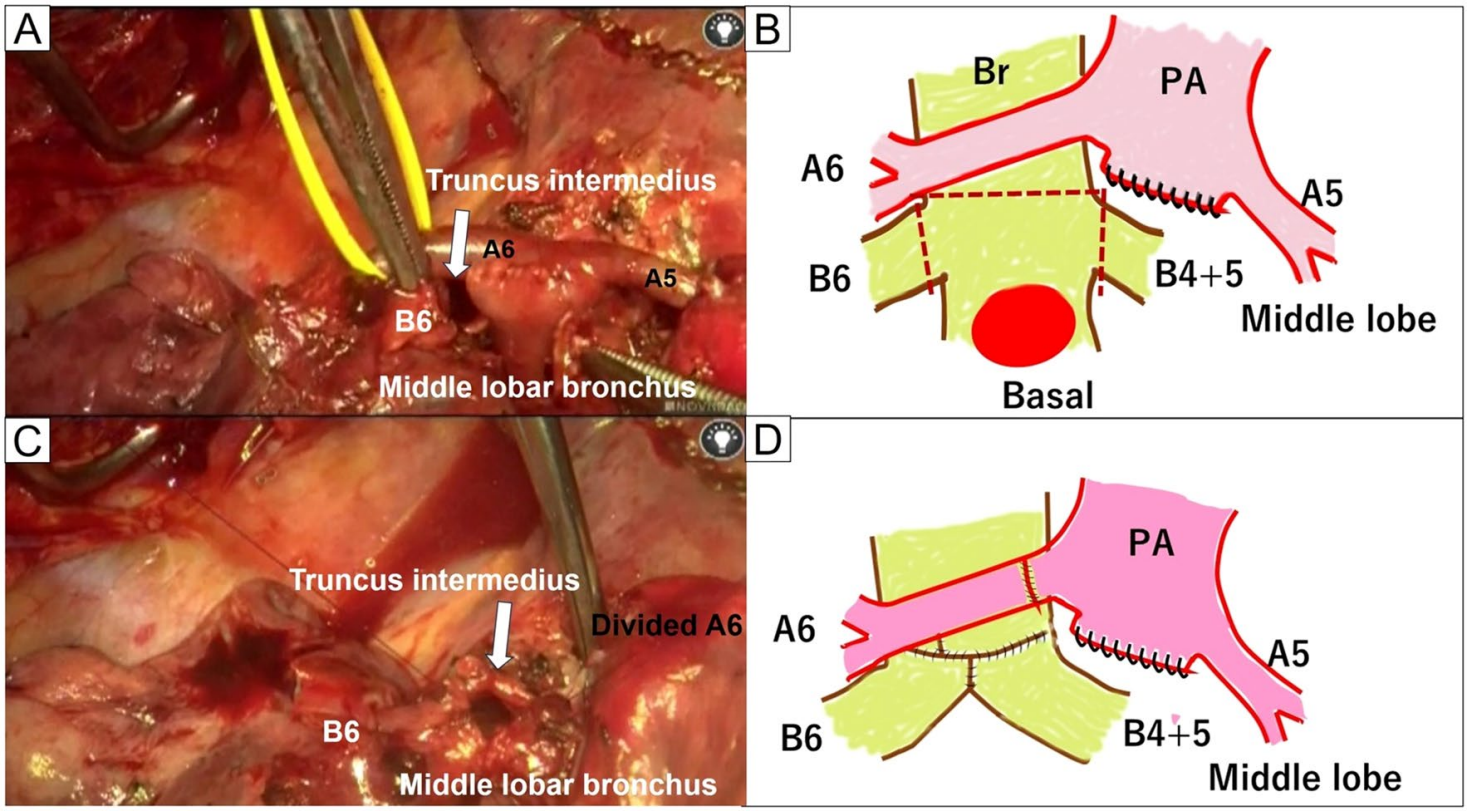
Intraoperative findings after bronchial resection. **A**, **B** A6 was divided after sleeve basal segmentectomy was performed. **C**, **D** Sufficient surgical exposure was achieved after division

After confirmation of a negative central bronchial margin using a frozen section, bronchial reconstruction with double-barrel anastomosis using B6 and the middle lobar bronchus was attempted. However, the anastomosis site was completely obstructed behind the A6. To ensure an adequate surgical view for bronchial suturing, A6 was divided after proximal clamping of the interlobar artery and distal clamping of V6 under systemic heparinization (2000 U bolus) (Fig. 3A, B). The deepest area, including the middle lobar bronchus and the bronchus intermedius, was anastomosed using a running 5–0 polydioxanone suture. Subsequently, B6 and the bronchus intermedius were anastomosed. A few adjusting tack sutures were placed in the membranous part of the bronchus intermedius to minimize caliber difference, allowing for successful completion of the double-barrel anastomosis using a running suture. All of the knots were tied externally (Fig. 3C, D). Once the double-barrel anastomosis was completed, A6 was reconstructed using a running 6–0 polypropylene suture. Reventilation resulted in prompt lung expansion. Finally, the bronchial anastomosis was covered with a pedicle fat pad. The total operative time was 318 min. The estimated blood loss volume was 170 mL.

### Postoperative course

**Fig. 4:**
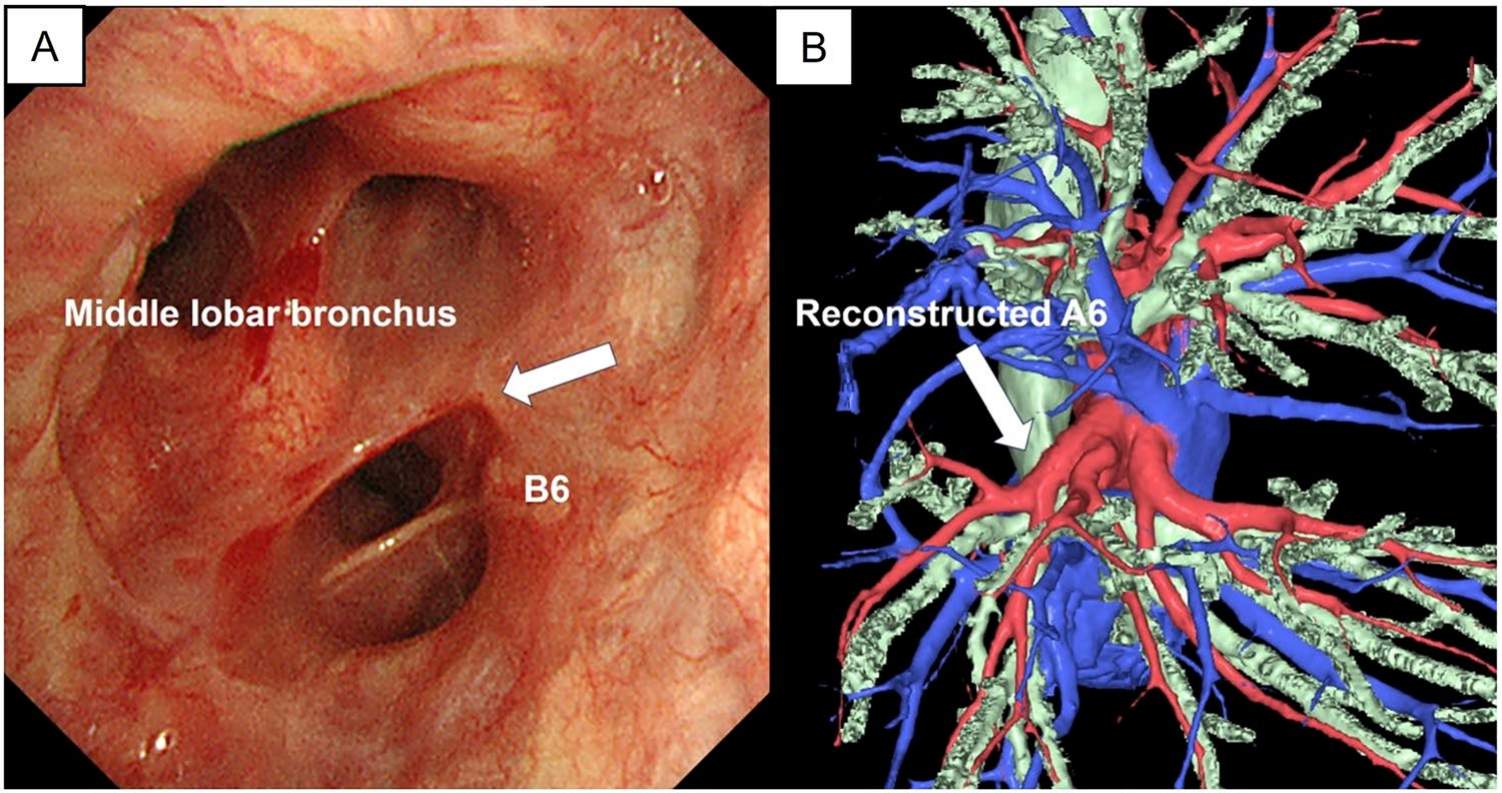
Postoperative findings. **A** Bronchoscopy performed 6 months after the surgery revealed excellent bronchial healing. **B** Contrast-enhanced computed tomography indicated sufficient A6 blood flow

The patient’s postoperative course was uneventful. After confirming adequate bronchial healing via bronchoscopy and CT on postoperative day 7, the chest drain was removed. The patient was discharged 3 days later. A postoperative histopathological examination revealed double squamous cell lung cancer with 12-mm surgical margins (S2:13 mm, pT1bN0; S7:28 mm, pT1cN0). At the 6-month follow-up, bronchoscopy showed excellent healing of the anastomosis without recurrence, and contrast-enhanced CT showed sufficient A6 blood flow (Fig. 4A, B, video).

## Discussion

The first sleeve resection was performed by Price-Thomas in a patient with a carcinoid tumor in 1947 [3]. Allison [4] used this technique to resect bronchogenic carcinomas in 1952. Bronchoplastic procedures have become widely used, because they can achieve survival rates similar to those of standard resection. Surgical decision-making in patients with lung malignancy must consider the preoperative lung function, the risks associated with each possible resection technique, and the risk of recurrence. In our immunosuppressed patient with a preserved lung function, although the risk of bronchial anastomosis-related complications seemed to be relatively high, intentional sleeve segmentectomy was performed. Although segmentectomy is feasible in patients with anatomically suitable tumors when it can provide survival outcomes equivalent to those of lobectomy, an intraoperative frozen pathological examination should be performed to confirm tumor-free surgical margins and determine the presence of lymph node metastasis.

Bronchoplasty at the segmental level was initially developed for early lung cancer and low-grade malignant tumors. However, segmental bronchoplasty is rarely indicated, because most lung cancers cannot be completely removed using this technique [5]. Segmentectomy with double-barrel reconstruction is less frequently performed because of the presence of the surrounding pulmonary hilar vessels. Although we could not find a previous report describing sleeve basal segmentectomy with double-barrel reconstruction, six cases of double-barrel bronchoplasty in the segmental region for lung tumors have been reported [6–10].

There are several unique aspects of bronchial healing after segmental bronchial sleeve resection with double-barreled reconstruction. The anastomosis site requires minimal tension and adequate blood supply. Mobilization of peribronchial tissue and arteries is required to enable an adequate surgical view to perform bronchial reconstruction after resection. To maintain blood supply to the bronchial anastomosis in our patient, the pulmonary artery was divided without detachment of the surrounding peribronchial tissue and then reconstructed. Although bronchoscopy performed 3 months after surgery showed delayed healing of the bronchial anastomosis, healing and A6 blood flow were sufficient at the 6-month follow-up, indicating that our strategy was successful. Now that segmentectomy is being widely used for the treatment of lung cancer, thoracic surgeons must be able to perform it as well as the common types of bronchoplasty.

## Supplementary Information

Below is the link to the electronic supplementary material.

Supplementary file1 (MP4 27483 KB)

## Data Availability

All relevant data are available in the manuscript and the Supporting Information files.
